# The combination of dermoscopy and reflectance confocal microscopy increases the diagnostic confidence of amelanotic/hypomelanotic lentigo maligna

**DOI:** 10.1111/1346-8138.17075

**Published:** 2024-01-13

**Authors:** Maria Antonietta Pizzichetta, Jerry Polesel, Jean Luc Perrot, Pietro Rubegni, Ignazio Stanganelli, Serena Magi, Laura Mazzoni, Francesca Farnetani, Giovanni Pellacani, Mattia Garutti, Fabio Puglisi, Elisa Cinotti, the Italian Melanoma Intergroup, Società Italiana di Dermatologia Chirurgica, Oncologica, Correttiva ed Estetica, and Groupe d'imagerie cutanée non invasive de la Société Française de Dermatologie

**Affiliations:** ^1^ Department of Dermatology University of Trieste Trieste Italy; ^2^ Department of Medical Oncology Centro di Riferimento Oncologico di Aviano IRCCS, Istituto di ricovero e cura a carattere scientifico Aviano Italy; ^3^ Cancer Epidemiology Unit Centro di Riferimento Oncologico di Aviano IRCCS Aviano Italy; ^4^ Department of Dermatology University Hospital of Saint Etienne Saint‐Etienne France; ^5^ Department of Medical, Surgical and Neurological Science, Dermatology Section University of Siena, S. Maria alle Scotte Hospital Siena Italy; ^6^ Department of Dermatology University of Parma‐Skin Cancer Unit, Istituto Scientifico Romagnolo per lo Studio dei Tumori "Dino Amadori" IRCCS Meldola Italy; ^7^ Skin Cancer Unit IRCCS Istituto Romagnolo per lo Studio dei Tumori "Dino Amadori" Meldola Italy; ^8^ Department of Dermatology University of Modena and Reggio Emilia Modena Italy; ^9^ Department of Dermatology, Policlinico Umberto I Sapienza University of Rome Rome Italy; ^10^ Department of Medicine University of Udine Udine Italy

**Keywords:** amelanotic melanoma, dermoscopy, lentigo maligna, nonmelanocytic skin lesion reflectance confocal microscopy

## Abstract

The dermoscopic diagnosis of amelanotic/hypomelanotic lentigo maligna/lentigo maligna melanoma (AHLM/LMM) may be very difficult in its early stages because of lack of pigment. Reflectance confocal microscopy (RCM) is an imaging technique that is especially helpful for the diagnosis of lentigo maligna. To determine the diagnostic performances of dermoscopy and RCM in the diagnosis of AHLM/LMMs we evaluated dermoscopic and RCM images of consecutive cases of histopathologically confirmed AHLM/LMMs, amelanotic/hypomelanotic basal cell carcinoma and squamous cell carcinoma (AHBCCs/AHSCCs), amelanotic/hypomelanotic benign lesions (AHBLs), and actinic keratoses (AKs) from five participating centers. Sensitivity, specificity, accuracy, predictive values, and level of diagnosis confidence were calculated for both diagnostic procedures. Both dermoscopy and RCM showed diagnostic performance >97% in the diagnosis of AHLM/LMMs versus AHBCC/AHSCCs and their combination slightly improved diagnostic performance, with accuracy increasing from 98.0% to 99.1%. Similarly, RCM in combination with dermoscopy showed a tiny increase in the diagnostic performance in the diagnosis of AHLM/LMMs versus AHBLs (accuracy increased from 87.2% to 88.8%) and versus AKs (accuracy increased from 91.4% to 93.4%). Although the increase in diagnostic performance due to RCM was modest, the combination of dermoscopy and RCM greatly increased the level of confidence; high confidence in the diagnosis of AHLM/LMMs versus AHBLs increased from 36.2% with dermoscopy alone to 76.6% with dermoscopy plus RMC. Based on our results, dermoscopy and RCM should be complementary to improve not only diagnostic accuracy but also the level of diagnostic certainty in the diagnosis of AHLM/LMMs.

## 1 INTRODUCTION

Amelanotic/hypomelanotic lentigo maligna/lentigo maligna melanoma (AHLM/LMM) may be very difficult to diagnose in its early stages because of lack of pigment; it may mimic inflammatory lesions and benign or malignant nonmelanocytic lesions.^1^, ^2^ The literature on dermoscopic diagnosis for AHLM/LMM is scanty and reflectance confocal microscopy (RCM) is an imaging technique that is especially helpful for lesions on the head and neck, damaged by chronic sun exposure.^3^, ^4^, ^5^, ^6^

In this retrospective study, we revised the dermoscopic and RMC features of 224 consecutive amelanotic/ hypomelanotic (extent of pigmentation ≤25%) flat skin lesions of the head and neck to evaluate their diagnostic performances in the diagnosis of AHLM/LMMs.

## 2 PATIENTS AND METHODS

We collected consecutive cases of histopathologically confirmed AHLM/LMMs, amelanotic/hypomelanotic basal cell carcinoma and squamous cell carcinoma (AHBCCs/AHSCCs), amelanotic/hypomelanotic benign lesions (AHBLs), and actinic keratosis (AKs) from five participating centers between January 2010 and December 2019.^7^ Dermoscopic and RCM images of these cases were evaluated by a panel of three blinded observers; the dermoscopic, RCM, and dermoscopic plus RCM diagnoses were achieved when three of three or two of three observers agreed. For each case we evaluated two dermoscopic images and three to five RCM images; examination with a Vivascope 3000 was performed only by RCM experts on more relevant and representative lesion areas dermoscopically. The dermoscopic and RCM features evaluated were based on previously described features for LM/LMM, BCC, SCC, AK, solar lentigo, seborrhoeic keratosis, and liken‐planus keratosis.^3^, ^4^, ^6^, ^7^, ^8^, ^9^ The level of diagnosis confidence was assessed using a three‐level scale (i.e., low, medium, and high).

Diagnostic performances (i.e., sensitivity, specificity, accuracy, predictive values, and level of confidence) were calculated for each diagnostic procedure as a percentage, with corresponding 95% confidence intervals according to the Clopper–Pearson method, using histopathology as the gold standard. Differences in the proportion of a high level of confidence were evaluated through the McNemar test. Approval by the Board of Ethics is waived for retrospective studies based on the Italian research regulations (D.Lgs. 101/2018, art. 8) because patients give their consent to the use of clinical data for research purposes, including publication of photographic material, at hospitalization. The signed consent is kept, according to Italian regulations, under the responsibility of the principal investigator of each participating center.

## 3 RESULTS

Our study population consisted of 224 lesions in 216 patients (116 men, 100 women) with a median age of 67 years. The study included 55 AHLMM/LMMs, 62 AHBCC/AHSCCs, 56 AHBLs (solar lentigo, seborrhoeic keratosis, and liken‐planus like keratosis), and 51 AKs.^7^

The diagnostic performance and level of confidence for dermoscopy, RCM, and dermoscopy plus RCM in the diagnosis of AHLM/LMMs versus AHBLs, AHBCCs/AHSCCs, and AKs are reported in the Table 1. Both dermoscopy and RCM showed diagnostic performance >97% in the diagnosis of AHLM/LMMs versus AHBCC/AHSCCs and their combination slightly improved diagnostic performance, with accuracy increasing from 98.0% to 99.1%. Similarly, RCM in combination with dermoscopy showed a tiny increase in the diagnostic performance in the diagnosis of AHLM/LMMs versus AHBLs (accuracy increased from 87.2% to 88.8%) and versus AKs (accuracy increased from 91.4% to 93.4%). Although the increase in diagnostic performance due to RCM was modest, the combination of dermoscopy and RCM greatly increased the level of confidence. Indeed, high confidence in the diagnosis of AHLM/LMMs versus AHBLs increased from 36.2% with dermoscopy to 76.6% (*P* < 0.001) with dermoscopy plus RMC. High confidence in the diagnosis of AHLM/LMM versus AKs increased from 23.5% with dermoscopy to 66.3% with RCM alone (*P* = 0.005) and to 70.8% for dermoscopy plus RMC (*P* < 0.001).

**TABLE 1:** Diagnostic performance and level of confidence (percentage) of dermoscopic and RCM diagnosis in the diagnosis of AHLM/LMMs vs AHBLs, AHBCCs/AHSCCs, and AKs.

	Dermoscopy	RCM	Dermoscopy plus RCM
Diagnosis (*n*)			
AHLM/LMM	60	66	64
AHBCC/AHSCC	66	68	66
AHBL	57	54	49
AK	41	36	45
AHLM/LMMs vs AHBCC/AHSCCs			
Diagnostic performance			
Sensitivity	97.9%	100%	100%
Specificity	98.0%	98.2%	98.2%
Accuracy	98.0%	99.1%	99.1%
Positive predictive value	97.9%	98.2%	98.2%
Negative predictive value	98.0%	100%	100%
Level of confidence			
Low	24.5%	15.4%	8.6%
Medium	29.6%	12.0%	12.0%
High	45.9%	72.7%	79.5%
AHLM/LMMs vs AHBLs			
Diagnostic performance			
Sensitivity	90.4%	92.6%	92.7%
Specificity	83.3%	82.2%	83.7%
Accuracy	87.2%	87.9%	88.8%
Positive predictive value	87.0%	86.2%	87.9%
Negative predictive value	87.5%	90.2%	90.0%
Level of confidence			
Low	33.0%	12.1%	10.8%
Medium	30.9%	13.2%	12.6%
High	36.2%	71.7%	76.6%
AHLM/LMMs vs AKs			
Diagnostic performance			
Sensitivity	95.9%	98.0%	98.1%
Specificity	84.4%	80.0%	87.2%
Accuracy	91.4%	90.7%	93.4%
Positive predictive value	90.4%	87.7%	91.1%
Negative predictive value	93.1%	96.6%	97.1%
Level of confidence			
Low	35.8%	16.3%	14.2%
Medium	40.7%	17.4%	15.1%
High	23.5%	66.3%	70.8%

Abbreviations: AHBCC/AHSCC, amelanotic/hypomelanotic basal and squamous cell carcinoma; AHBL, amelanotic/hypomelanotic benign lesion; AHLM/LMM, amelanotic/hypomelanotic lentigo maligna/lentigo maligna melanoma; AK, actinic keratosis; RCM, Reflectance confocal microscopy

## 4 DISCUSSION

**FIGURE 1:**
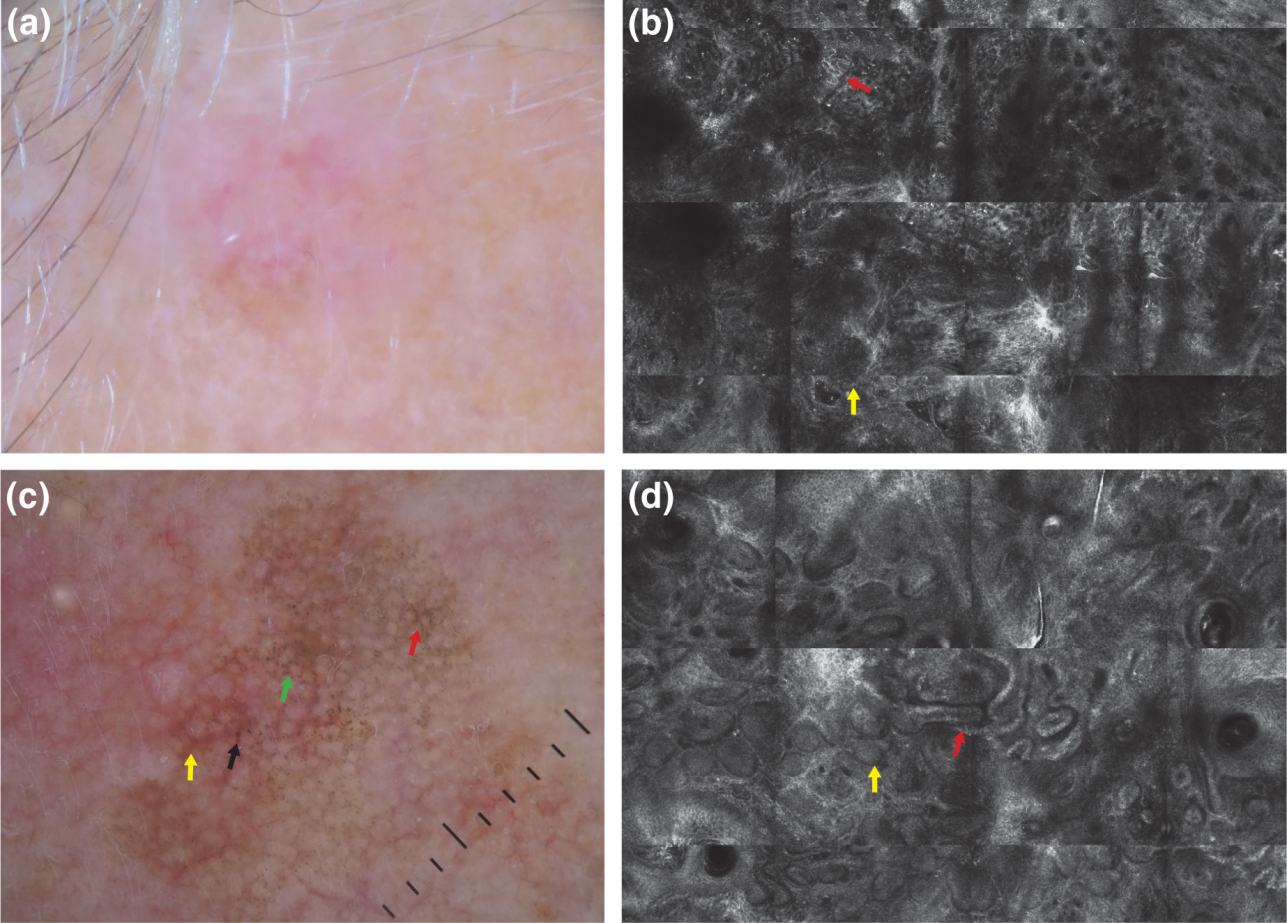
(a, b) Dermoscopic and reflectance confocal microscopy (RCM) images of a non‐pigmented basal cell carcinoma on the forehead of a 62‐year‐old woman. (c, d) Dermoscopic and RCM images of an actinic keratosis on the cheek of a 52‐year‐old woman. (a) Dermoscopically, the lesion reveals unfocused multiple shades of pink and a peripheral light‐brown structureless area, difficult to differentiate from a melanocytic lesion (10× magnification). (b) RCM performed with a VivaScope 1500 (Mavig, Munich) reveals dermis tumor cords with peripheral palisading (red arrow) and an island (yellow arrow), the hallmark for BCC diagnosis on RCM. (c) In the dermoscopic image, asymmetric pigmented follicles (black arrow), annular granular structures around follicles (green arrow), rhomboidal structures (red arrow), and red rhomboidal structures (yellow arrow) can be seen. (d) RCM did not show any atypical melanocytes at the epidermal layer, but polycyclic papillary contours of the papillae (red arrow) and bulbous projections (yellow arrow) can be seen at the dermo epidermal junction.

Based on our results, the combination of dermoscopy and RCM showed higher diagnostic performance for the diagnosis of AHLM/LMMs versus AHBCC/AHSCCs rather than vesus AHBLs and AKs. Sometimes AHBCC may be difficult to differentiate from AHLM/LMM dermoscopically because of multiple shades of pink and peripheral light‐brown structureless areas (Figure 1a), but in RCM the detection of dermis tumor cords with peripheral palisading and island (Figure 1b), a hallmark for BCC diagnosis on RCM,^10^ makes the discrimination between AHLM/LMM and AHBCC easier.

Although in our study the increase in accuracy due to dermoscopy plus RCM for the diagnosis of AHLM/LMMs versus AHBLs was modest, the combination of the two techniques considerably increased the level of confidence in the diagnosis of AHLM/LMMs versus AHBLs and AHLM/LMMs versus AKs.

Dermoscopically, AK may share with LM some features such as annular‐granular pattern, rhomboidal structures, and asymmetric pigmented follicular openings^8^, ^11^ (Figure 1c). In these equivocal lesions RCM may reveal features associated with benign lesions as polycyclic papillary contours of the papillae and bulbous projections (Figure 1d), making a more confident diagnosis without a biopsy confirming the diagnosis. This may have relevant clinical implications, since a more confident diagnosis may reduce the number of equivocal lesions to address to partial biopsy or surgical excision.

The high level of confidence with dermoscopy plus RCM in the diagnosis of AHLM/LMMs versus AKs was lower (70.8%) than in the diagnosis of AHLM/LMMs versus AHBLs (76.6%). The confounding RCM feature in AK was the frequent detection of intraepidermal Langherans dendritic cells, which can be challenging to differentiate from melanocytic dendritic cells, one of the relevant criteria for melanoma diagnosis.^12^ This could explain why it may be difficult to differentiate AHLM/LMM from AK in RCM.

**FIGURE 2:**
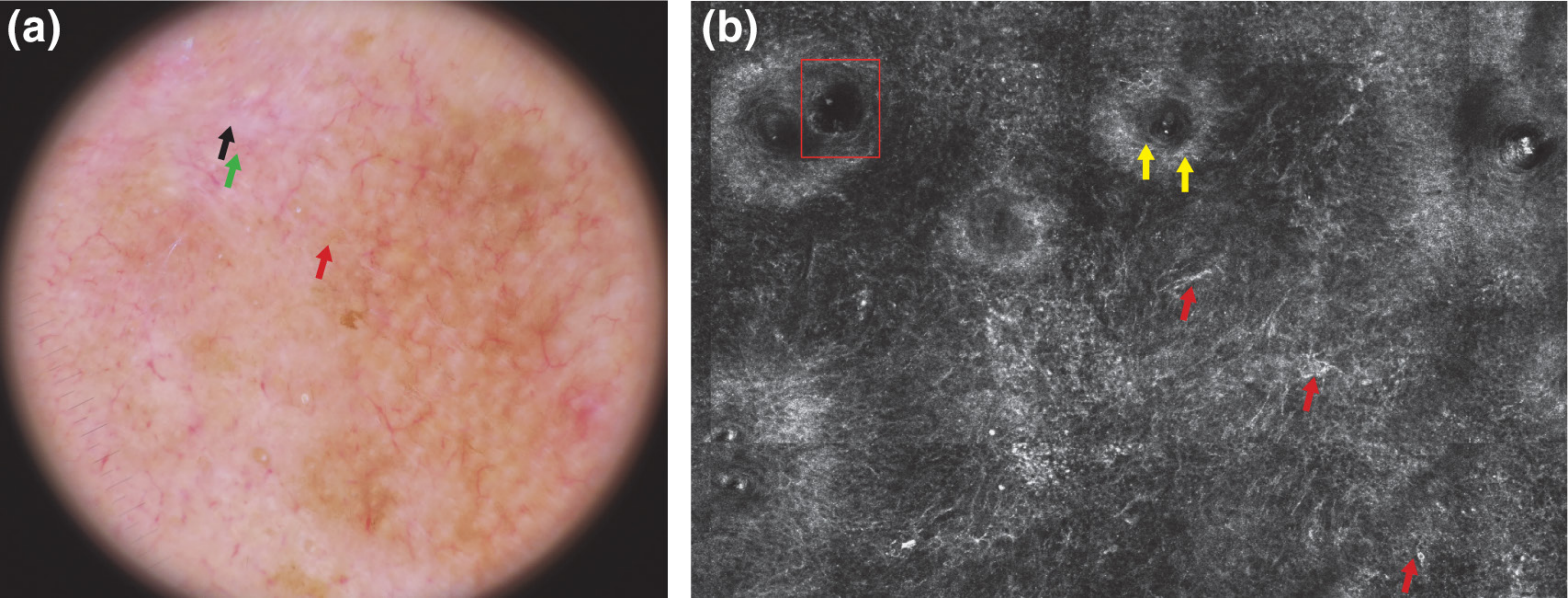
Dermoscopic and reflectance confocal microscopy (RCM) images of an amelanotic lentigo maligna on the cheek of a 58‐year‐old man. (a) At dermoscopic examination, the lesion was characterized by an irregular brown pigmentation, annular granular structures (red arrow), shiny with structures (black arrow), and linear irregular vessels (green arrow). (b) On RCM examination, atypical large hyper‐reflective dendritic junctional cells among hair follicles (red arrows), follicular localization of dendritic cells (yellow arrows), and hyporeflective nests in perifollicular distribution (red rectangle) were detectable at the dermo epidermal junction.

Dermoscopically, early LM may be difficult to identify because it may share similar features with actinic keratosis or solar lentigo^8^; differentiating AHLM/LMMs from actinic keratosis or solar lentigo on dermoscopy may be challenging (Figure 2a). In difficult equivocal amelanotic/hypomelanotic lesions, the integration of dermoscopy with RCM greatly increased the level of diagnostic confidence in the diagnosis of AHLM/LMMs thanks to the visualization on RCM of features associated with of AHLM/LMMs, such as large hyper‐reflective dendritic junctional cells, focal follicular localization of dendritic cells, and hyporeflective nests in perifollicular distribution^6^, ^7^ (Figure 2b). Furthermore, the combination of dermoscopy and RCM could improve the diagnosis of AHLM/LMMs because dermoscopically suspicious lesions are observed with more attention at RCM because careful zooming of the images is essential to identify hyporeflective pagetoid cells, as we have described previously for amelanotic/hypomelanotic melanoma not of LM subtype.^13^ Notably, RCM can be especially helpful for amelanotic/hypomelanotic melanomas because they have melanosomes and rare melanin granules that appear bright under RCM even if they are not dermoscopically visible.^6^

In our study, dermoscopy and RCM were similar in terms of specificity (92% vs 94%, respectively). These diagnostic performances are quite different from those reported by Cinotti et al.,^14^, ^15^ who found that RCM was more sensitive and less specific than dermoscopy for LM diagnosis versus other benign and malignant lesions; they reported sensitivity of 80% and 61% and specificity of 81% and 92% for RCM and dermoscopy, respectively.^14^ The differences between the two studies could result from the fact that in the study by Cinotti et al.^14^ the RCM evaluation was performed in blind to dermoscopy In addition, this latter study also included pigmented lesions with only 17 hypomelanotic and one amelanotic cases. However, in this latter study RCM had a higher sensitivity (69%) than dermoscopy (37%) for hypomelanotic LM/LMMs.^14^

This study shows that the integration of dermoscopy and RCM increases the level of diagnostic certainty in the diagnosis of AHLM/LMMs versus AHBCCs/AHSCCs, AHBLs, and AKs compared to dermoscopy and RCM alone. This is particularly important for AHBLs and AKs because besides increasing the sensitivity in diagnosing AHLM/LMMs, the combination of the two techniques can reduce the number of dermoscopically equivocal lesions to address to biopsy or surgical excision to confirm the diagnosis. In conclusion, dermoscopy and RCM should be complementary to improve not only diagnostic accuracy, but also the level of diagnostic certainty in the diagnosis of AHLM/LMMs, a difficult‐to‐diagnose subtype of melanoma.

## FUNDING INFORMATION

This work was partially supported by the Italian Ministry of Health (Ricerca Corrente) (no grant number provided).

## CONFLICT OF INTEREST STATEMENT

None declared.
